# Eryptosis: Programmed Death of Nucleus-Free, Iron-Filled Blood Cells

**DOI:** 10.3390/cells11030503

**Published:** 2022-02-01

**Authors:** Peter Dreischer, Michael Duszenko, Jasmin Stein, Thomas Wieder

**Affiliations:** 1 Abteilung für Vegetative und Klinische Physiologie, Physiologisches Institut, Universität Tübingen, 72074 Tübingen, Germany; p.dreischer@uni-tuebingen.de; 2 Abteilung für Hebammenwissenschaft, Institut für Gesundheitswissenschaften, Universität Tübingen, 72074 Tübingen, Germany; 3 Interfakultäres Institut für Biochemie, Universität Tübingen, 72076 Tübingen, Germany; michael.duszenko@uni-tuebingen.de (M.D.); Jasmin_Stein@gmx.de (J.S.); 4 Universitätsklinikum Tübingen, Universitäts-Hautklinik, 72076 Tübingen, Germany

**Keywords:** anaerobic metabolism, anemia, cell volume, cytoskeleton, DNA damage, oxidative stress, ferroptosis, phosphatidylserine exposure, programmed cell death, protease activation

## Abstract

Human erythrocytes are organelle-free cells packaged with iron-containing hemoglobin, specializing in the transport of oxygen. With a total number of approximately 25 trillion cells per individual, the erythrocyte is the most abundant cell type not only in blood but in the whole organism. Despite their low complexity and their inability to transcriptionally upregulate antioxidant defense mechanisms, they display a relatively long life time, of 120 days. This ensures the maintenance of tissue homeostasis where the clearance of old or damaged erythrocytes is kept in balance with erythropoiesis. Whereas the regulatory mechanisms of erythropoiesis have been elucidated over decades of intensive research, the understanding of the mechanisms of erythrocyte clearance still requires some refinement. Here, we present the main pathways leading to eryptosis, the programmed death of erythrocytes, with special emphasis on Ca^2+^ influx, the generation of ceramide, oxidative stress, kinase activation, and iron metabolism. We also compare stress-induced erythrocyte death with erythrocyte ageing and clearance, and discuss the similarities between eryptosis and ferroptosis, the iron-dependent regulated death of nucleated blood cells. Finally, we focus on the pathologic consequences of deranged eryptosis, and discuss eryptosis in the context of different infectious diseases, e.g., viral or parasitic infections, and hematologic disorders.

## 1. Introduction

Erythrocytes are organelle-free cells possessing huge amounts of iron-containing tetrameric hemoglobin (Hb). Depending on the partial oxygen pressure, each Hb molecule binds or releases four molecules of oxygen (O_2_). Thus, erythrocytes are perfectly designed to fulfill their main function, i.e., the transport of oxygen (O_2_) from the lungs to the organs [1]. Besides this, they also play a crucial role in transporting other breathing gases, such as carbon dioxide (CO_2_) [2]. As the Hb molecule switches its conformation between the oxy (R) and deoxy (T) state during the erythrocyte’s arterial-venous passage, it delivers O_2_ and takes up CO_2_ and protons (H^+^) in tissue capillaries. This process is elegantly supported by the Bohr effect, i.e., a decreased oxygen affinity due to an increase in acidity and CO_2_ concentration. In addition, erythrocytes contain carbonic anhydrase, an enzyme with one of the highest turnover numbers, thus providing fast hydration of metabolic CO_2_. After the dissociation of the reaction product carbonic acid (H_2_CO_3_), H^+^ is bound to Hb (Haldane effect) while bicarbonate (HCO_3_^-^) is shifted into the plasma via an anion exchanger.

**Figure 1:**
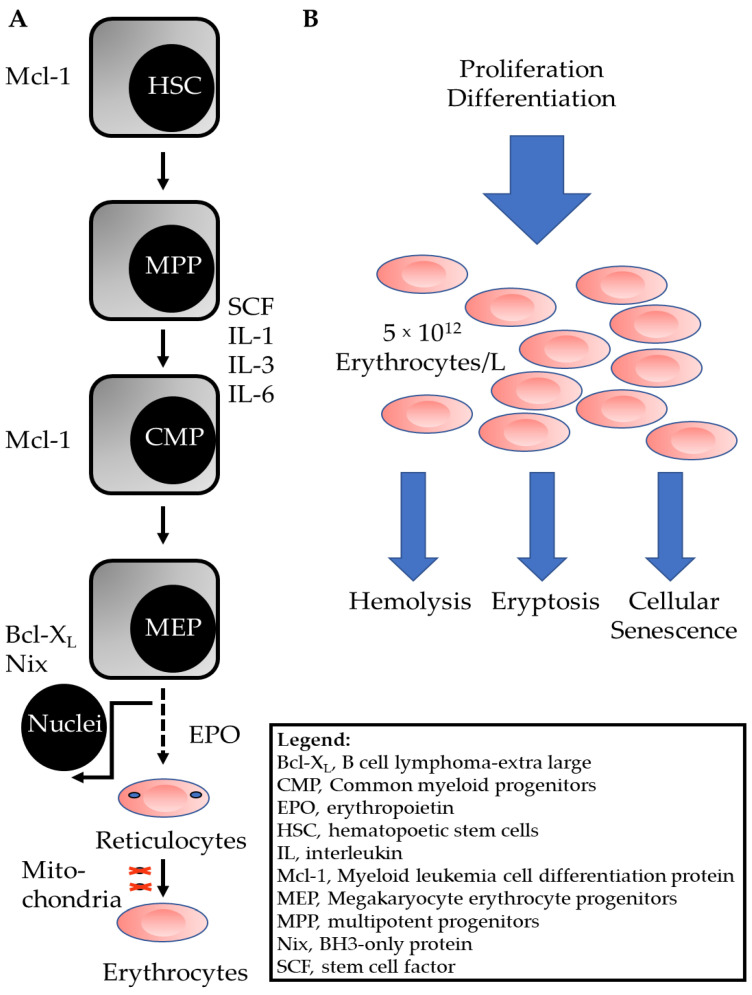
Scheme of erythrocyte generation and differentiation in the bone marrow, and erythrocyte homeostasis. (**A**) The scheme depicts the development of erythrocyte progenitor cells in the red bone marrow [3]. Death-regulating intracellular proteins are shown on the left and extracellular growth or differentiation factors are shown on the right. The development of white blood cells from CMP is not shown. (**B**) The pool of mature erythrocytes is fueled by proliferation and differentiation as outlined in (**A**) Old or damaged cells are removed from the pool by hemolysis, eryptosis or cellular senescence.

The various vertebrate blood cell types, such as erythrocytes, thrombocytes, granulocytes, lymphocytes, originate from common progenitor cells in the bone marrow (for review see [3], and references specified therein). These self-renewing cells, so-called hematopoietic stem cells (HSC), give rise to multipotent progenitor cells (MPP), as outlined in Figure 1A. Under the control of stem cell factor (SCF) and interleukins (IL)-1, IL-3, and IL-6, common myeloid progenitors (CMP) are generated that develop into megakaryocyte erythrocyte progenitors (MEP). The specific growth factor erythropoietin (EPO) then triggers the proliferation of erythroid progenitor cells (burst-forming unit-erythroid). Even more importantly, EPO induces the differentiation and enucleation of the resulting cells of the colony-forming unit-erythroid (CFU-e) [4]. As a result of the enucleation of CFU-e cells and phagocytosis of the nuclei by macrophages [5], nuclei-free reticulocytes are formed (Figure 1A). In the last step of the differentiation process, reticulocytes lose their remaining organelles, such as mitochondria, mainly by autophagic processes [6].

To meet the body’s demand for O_2_, the huge number of cells in the pool of functional erythrocytes is kept at a constant level. This is achieved by the adjusted proliferation and differentiation of the progenitor cells, thereby replacing the senescent cells that had been removed from the pool, or the damaged erythrocytes that died by hemolysis or eryptosis (Figure 1B).

Mature human erythrocytes display a life span of approximately 120 days [7] before being removed from circulation. The relatively long life span of these cells is explained by two mechanisms: (i) during the last two stages of differentiation, the cells extrude two important organelles which play crucial roles in apoptosis pathways, i.e., the nucleus and the mitochondrion, and (ii) the cells express high levels of antiapoptotic proteins, such as myeloid leukemia cell differentiation protein (Mcl-1) and B cell lymphoma-extra-large (Bcl-X_L_). These proteins are already expressed during the different developmental stages of erythropoiesis (Figure 1A, [3]).

## 2. Pathways of Programmed Erythrocyte Death

About one percent of the human erythrocyte pool of 25 × 10^12^ cells per individual, which means approximately 250 × 10^9^ cells, must be removed from the circulation every day. As erythrocytes lose their organelles during the differentiation process, they are unable to proliferate and can thus be considered senescent. Consequently, they should not undergo classical apoptosis. It was therefore assumed that erythrocytes follow a predetermined aging process with regulated clearing mechanisms to remove old cells. Indeed, in the 1980s, conformational changes within the membrane domain of band 3, one of the most prominent proteins of the erythrocyte membrane, were discovered. These modified proteins are recognized by specific, autologous antibodies and are thus described as senescent cell-specific antigens [8,9]. Similarly to opsonized bacteria, old erythrocytes are tagged with specific antibodies for removal by macrophages [10]. The underlying mechanisms of erythrocyte aging were studied in vitro and in vivo, showing that senescence-associated changes in membrane lipids and proteins are mainly caused by oxidative stress, either directly or by hemoglobin denaturation [8,9,10,11,12].

In 2001, the ground-breaking discovery that ionomycin-mediated Ca^2+^-influx into mature erythrocytes induced the rapid self-destruction process of these cells [13,14] challenged the dogma that erythrocytes are unable to undergo programmed cell death. It was shown that ionomycin treatment of erythrocytes led to cell shrinkage, plasma membrane microvesiculation (blebbing), and phosphatidylserine externalization, all features of apoptotic cells. However, the dying cells did not activate caspases but, instead, the cysteine protease calpain [14]. Berg et al. therefore concluded that erythrocytes do not contain all the components of a functional system to execute apoptosis but may express proteins that mediate delayed cellular senescence. One year later, Lang et al. demonstrated that (i) physiological stimuli such as hyperosmotic shock and oxidative stress induce programmed cell death in mature erythrocytes [15] and (ii) the erythrocytes of patients suffering from sickle cell anemia, thalassemia or glucose-6-phosphate dehydrogenase deficiency are more susceptible to these physiological stimuli [16]. Nevertheless, the discussion over whether programmed erythrocyte death and erythrocyte senescence describe different cell death mechanisms or simply the same phenomenon continued. In 2005, the term eryptosis was coined [17], and the pathways and factors of programmed erythrocyte death were deciphered (see also Section 2.1, Section 2.2, Section 2.3, Section 2.4, Section 2.5 and Section 2.6). The most convincing argument that programmed erythrocyte death and erythrocyte senescence are two distinct pathways is the variation in their time constant of clearance: whereas stressed, eryptotic cells are cleared from the circulation in minutes [18], senescent erythrocytes are cleared in days [19].

**Figure 2:**
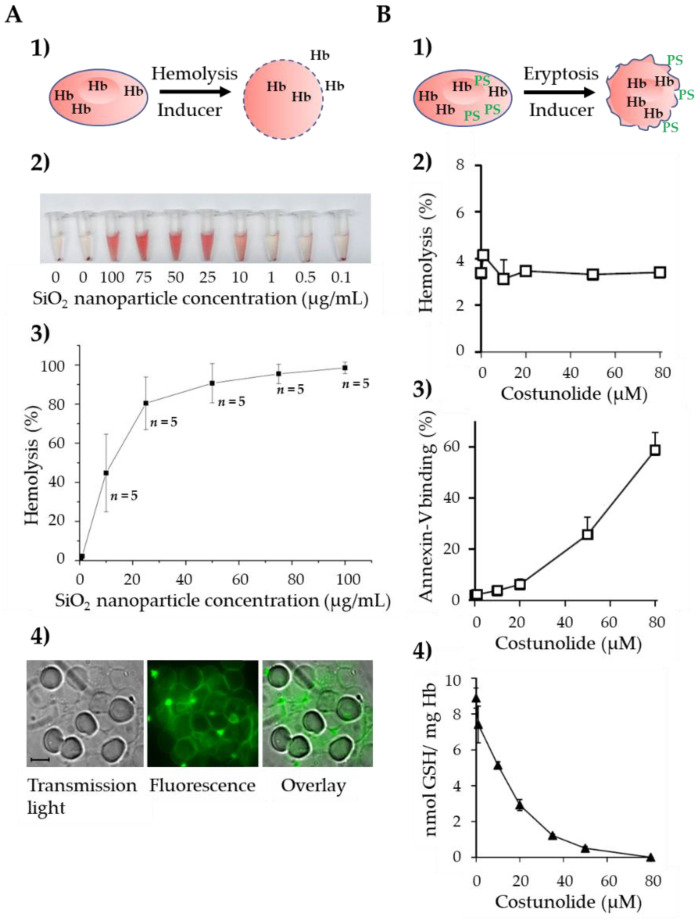
The two fundamental pathways of erythrocyte cell death. (**A**) Inducers of hemolysis lead to destruction of the cell membrane, erythrocytes lose their biconcave shape, and Hb is released to the extracellular space (**1**). Induction of concentration-dependent hemolysis by SiO_2_ nanoparticles. Hb release is demonstrated by the appearance of red-colored Hb in the supernatant (**2**) and quantified photometrically. Data are given in % ± SEM, *n* = 5 (**3**). Staining of erythrocytes with fluorescence-labeled SiO_2_ nanoparticles. Note that the nanoparticles are incorporated into the erythrocyte plasma membrane thereby destroying the phospholipid bilayer (**4**). (**B**) Inducers of eryptosis lead to cell shrinkage and dissipation of phosphatidylserine asymmetry. Note that the cell membrane stays intact with Hb remaining inside the stressed erythrocytes (**1**). Absence of substantial hemolysis after incubation with different concentrations of costunolide (**2**). Concentration-dependent increase of annexin-V-positive erythrocytes by costunolide, i.e., erythrocytes exposing phosphatidylserine on the outer leaflet of the plasma membrane (**3**), and concomitant decrease of the GSH level in costunolide-treated erythrocytes (**4**). The data shown in (**B**) are from [20]. Abbreviations: GSH, glutathione; Hb, hemoglobin; PS, phosphatidylserine; SiO_2_, silicon dioxide.

In contrast to classical hemolysis, which leads to destruction of the plasma membrane and the subsequent release of hemoglobin into the extracellular space, the plasma membrane of eryptotic cells remains intact, and hemoglobin is retained in the cytoplasm (Figure 2(A1,B1)). For example, the incubation of human erythrocytes with SiO_2_ nanoparticles induces the concentration-dependent lysis of the cells (Figure 2(A2)) with an ED_50_ of approximately 10 µg/mL (Figure 2(A3)). Staining of the erythrocytes with fluorescence-labeled SiO_2_ nanoparticles revealed that the nanoparticles were incorporated into the plasma membrane (Figure 2(A4)). On the other hand, the natural sesquiterpene lactone costunolide strongly enhances phosphatidylserine exposure in the absence of substantial hemoglobin release (Figure 2(B2,B3)), [20]). The presumed drug target is the cytosolic enzyme glucose-6-phosphate dehydrogenase (G6PDH), which is strongly inhibited by costunolide [20], thereby leading to the depletion of intracellular glutathione (Figure 2(B4)), [20]). Interestingly, LaRocca et al. showed that the binding of pore-forming toxins to human CD59 (hCD59) may trigger a third pathway of red blood cell (RBC) death, which shares key molecular factors with nucleated cell necroptosis, including dependence on Fas/FasL signaling and Receptor-Interacting Protein 1 (RIP1) phosphorylation. Thus, they termed this kind of erythrocyte death “RBC programmed necrosis” [21]. In the following subchapters, we focus on eryptosis and describe the mechanisms involved in the signaling and regulation of this important death pathway.

### 2.1. Ca^2+^-Induced Eryptosis

Programmed erythrocyte death was first described in the context of increased Ca^2+^ levels in the cytosol of cells [13,14]. In these studies, the authors used the ionophor ionomycin to artificially increase the cytosolic Ca^2+^ concentration. However, the question of whether an increase of cytosolic Ca^2+^ could also be induced by a channel-based mechanism remained enigmatic. In 2003, it was then shown by patch clamp technique that hyperosmotic shock and oxidative stress, two well-known triggers of programmed cell death in nucleated cells, activate Ca^2+^-permeable cation channels, thereby increasing the intracellular Ca^2+^ concentration [15]. The channels were further characterized, and it was shown that these nonselective cation channels can be activated by prostaglandin E_2_ (PGE_2_) [22] and inhibited by erythropoietin [23]. As a result, increased Ca^2+^ levels induce phosphatidylserine exposure and erythrocyte shrinkage [15]. The shrinkage of erythrocytes is mainly due to activation of Ca^2+^-dependent K^+^ channels [24], the so-called Gardos channels. It was shown that ionomycin-mediated increases in intracellular Ca^2+^ activate inwardly rectifying K^+^-selective channels in the erythrocyte membrane, leading to a significant decrease in cell volume. Interestingly, the Gardos K^+^ channel blockers charybdotoxin and clotrimazole not only prevented cell shrinkage but, at the same time, blunted Ca^2+^-mediated phosphatidylserine exposure [25]. Thus, Ca^2+^-sensitive K^+^ channels in erythrocytes are actively involved in the regulation of eryptosis. Future studies on cell volume and channel regulation by gaseous substances such as NO [26] or H_2_S [27] will lead to new insights into the molecular mechanisms leading to the accelerated death of this cell type.

### 2.2. Ceramide-Induced Eryptosis

Sphingolipids are integral components of the erythrocyte membrane, with sphingomyelin as the most prominent member of this lipid class. It takes part in cell signaling in different tissues, such as the skin. Ceramides, as products of agonist-stimulated sphingomyelin hydrolysis, have been described as second messengers of the sphingomyelinase-driven ‘sphingomyelin cycle’ [28]. Ceramides induce cell differentiation, apoptosis, and senescence [28,29,30]. They are visualized by antibody-based fluorescence staining and quantitatively measured by flow cytometry [31]. The observation that hyperosmotic shock-induced eryptosis is only partly dependent on Ca^2+^ entry pointed to additional mechanisms. Indeed, Lang et al. demonstrated that ceramide is another important second messenger leading to programmed erythrocyte death [32]. Their study showed that (i) ceramides are formed following sphingomyelin breakdown after challenge of erythrocytes with hyperosmotic solutions, (ii) hyperosmotic shock-induced eryptosis is blunted by inhibitors of sphingomyelinase, and (iii) the proeryptotic effects of hyperosmotic shock can be mimicked by cell-membrane-permeable ceramides or by the addition of sphingomyelinase [32]. Further studies revealed that ceramide formation is regulated by platelet activating factor (PAF), which, after binding to its receptor in the erythrocyte membrane, activates the sphingomyelinase, thereby increasing intracellular ceramide levels [33]. Besides hyperosmotic shock and PAF, recent work established ceramide as a central regulator of eryptosis after the treatment of erythrocytes with Cu^2+^ [34], the *Pseudomonas aeruginosa* virulence factor pyocyanin [35] or the endogenous signaling molecule 4-hydroxy-trans-2-nonenal (HNE) [36]. Thus, ceramide together with Ca^2+^ entry from extracellular space combine to trigger erythrocyte death during different physiological and pathophysiological conditions.

### 2.3. Oxidative Stress-Induced Eryptosis

Erythrocytes transport huge amounts of O_2_ and are therefore subject to permanent oxidative stress. To counteract this pressure, erythrocytes contain high amounts of antioxidative glutathione (GSH) [37]. Nevertheless, it became clear in the very beginning of eryptosis research that overriding the antioxidative defense of erythrocytes by treating the cells with tert-butyl-hydroperoxide is one of the most powerful triggers of phosphatidylserine exposure and erythrocyte shrinkage [15]. The exact signaling pathways of oxidative-stress-induced eryptosis are still not fully understood, but there is crosstalk between Ca^2+^ channel activation, GSH levels, and reactive oxygen species [38,39,40]. Interestingly, directly targeting GSH metabolism using costunolide or dimethyl fumarate [20,37,41], which inhibit glucose-6-phosphate dehydrogenase activity [41], is a strong trigger of eryptosis.

### 2.4. Role of Protein Kinases

As signaling by protein kinases mainly takes place in the cytoplasm, it is likely that considerable parts of these well characterized pathways play a role in erythrocytes. Indeed, in 2002, the protein kinase C-dependent omega-agatoxin-TK-sensitive Ca^2+^ permeability of the erythrocyte membrane was described [42]. The stimulation of protein kinase C (PKC) using phorbol esters led to the opening of Ca(v) 2.1 channels, Ca^2+^ entry into erythrocytes and, later on, to phospholipid scrambling and cell shrinkage, that is, to eryptosis [43]. Thus, in contrast to nucleated cells, the activation of PKC kills erythrocytes, whereas the inhibition of PKC protects them [20]. Further studies revealed that the activation of erythrocyte PKC can also be induced by glucose depletion [44], thereby playing a pivotal role in certain pathophysiological situations. Recently, kinomics data on erythrocytes were published, demonstrating that they give rise to a complex kinase network that includes the non-receptor tyrosine kinase Src, the focal adhesion kinase FAK, the protein kinases C α and δ, the serine/threonine kinase ERK1, and others [45,46]. In the context of eryptosis, it has been shown that p38 kinase, PKC, Janus-activated kinase 3, casein kinase 1α, and cyclin-dependent kinase 4 stimulate erythrocyte death, whereas AMP-activated kinase, p21-activated kinase 2, cGMP-dependent protein kinase, mitogen- and stress-activated kinase MSK1/2, and some ill-defined tyrosine kinases inhibit eryptosis (for a review, see [47] and the references therein).

### 2.5. Role of Proteases

During the early differentiation process, effector caspases, such as caspase-3, are transiently activated in erythroid progenitor cells [48]. It was demonstrated that caspase activation also occurs in erythroid progenitor cells that have been challenged with interferon-γ, thereby driving cells into apoptosis [49]. In 2001, Western blot analyses revealed that mature human erythrocytes contain different pro-caspases, such as pro-caspase-3 and pro-caspase-8. However, the pro-caspases could not be activated by ionomycin or prolonged storage [14], nor by proeryptotic hyperosmotic shock [32]. Caspase activation in erythrocytes has only been described after oxidative stress and after FasL/Fas ligation [50,51]. On the other hand, increased intracellular Ca^2+^ levels and other stimuli, such as prostaglandins (PGE_2_), led to the activation of the cysteine protease calpain, which mediated fodrin cleavage or ankyrin-R degradation [14,22]. Calpain activation in the context of erythrocyte death has been repeatedly reported by different groups [52,53].

### 2.6. Anti-Eryptotic Factors

Erythrocytes are exposed to constant stress. With every passage across the renal medulla, the cells experience huge changes to their hyperosmotic conditions, reaching up to 1200 mosmol/L, while in the lungs, they have to withstand oxidative stress due to the high oxygen pressure. Thus, these cells need some defense mechanisms to keep them alive and prevent their premature clearance. In this regard, it has been shown that erythropoietin inhibits cation channels, thereby preventing eryptosis [23]. Further studies revealed that erythropoietin protects erythrocytes against oxidative stress [54,55], and the hormone may be useful for the treatment of patients suffering from decompensated autoimmune hemolytic anemia [56]. Another extracellular signaling molecule that regulates eryptosis is the gaseous transmitter nitric oxide (NO). In the vascular endothelium, NO is synthesized by the endothelial NO synthase (eNOS) from L-arginine and molecular oxygen. It can easily penetrate red blood cells by diffusion. In addition, NO can be directly formed and stored within erythrocytes. More importantly, it has been demonstrated that NO is a ubiquitous inhibitor that counteracts nearly all the triggers of eryptosis, such as ionomycin, glucose depletion, and hyperosmotic shock [57], oxidative stress [58], or trifluoperazine [59]. Besides the extracellular, anti-eryptotic factors mentioned above, Ghashghaeinia et al. demonstrated that red blood cells contain nuclear factor κB (NFκB) [60], a transcription factor with pro-survival activities in nucleated cells. The use of specific NFκB inhibitors drove erythrocytes into programmed cell death, thereby further pointing to their anti-eryptotic function [60]. Interestingly, the role of the three anti-eryptotic factors, erythropoietin, NO synthase, and NFκB, has been investigated in clinical settings, i.e., in autoimmune hemolytic anemia [56] and in obesity [61]. NFκB is not only involved in the regulation of eryptosis but also in the regulation of red blood cell senescence. In this respect, it was shown that the NFκB concentration dramatically declines during the aging of erythrocytes, which may be linked to the fact that spontaneous eryptosis is highest in aged erythrocytes [62].

**Figure 3:**
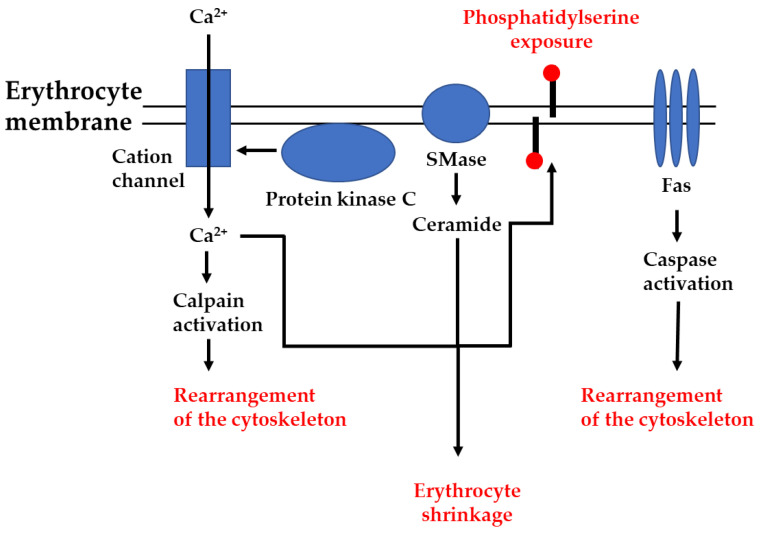
The main signaling pathways of eryptosis. Different inducers of eryptosis either lead to opening of cation channels and subsequent entry of Ca^2+^, activation of protein kinase C and subsequent opening of cation channels, activation of sphingomyelinase (SMase) and subsequent generation of ceramide, or clustering of death receptors (Fas) and subsequent activation of caspases. Ca^2+^ and ceramide combine to induce phosphatidylserine exposure and erythrocyte shrinkage, whereas the activation of proteases (caspases and calpain) leads to rearrangement of the cytoskeleton.

In summary, old or damaged red blood cells are removed from the circulation in three ways. Besides the unregulated hemolytic destruction of erythrocytes, there are two regulated mechanisms to remove these cells: (i) senescence, the physiological aging of mature erythrocytes; and (ii) eryptosis, non-hemolytic, stress-induced erythrocyte death. As eryptosis is a regulated process resembling apoptosis (programmed cell death), it is mandatory that erythrocytes express various signaling components and functional proteins that ensure the execution of the pathway. Figure 3 depicts the main signaling pathways of eryptosis leading to phosphatidylserine exposure, erythrocyte shrinkage, and the rearrangement of the cytoskeleton, and Table 1 summarizes important proteins of the eryptotic machinery that have already been detected in mature red blood cells.

## 3. Ferroptosis and Eryptosis: Similarities and Differences

In 2004, a new class of toxic, iron-containing nucleoside analogue was described [63] and its structure defined [64]. As these substances, in addition to iron atoms, contained nucleoside moieties, it was not surprising that they induced apoptosis in cancer cells, including DNA fragmentation [63], the activation of caspase-3 [64], and the elevation of reactive oxygen species (ROS) [65]. Nevertheless, the exact death pathway of this “iron-that-kills” remained to be elucidated. This took place in 2012, when Dixon et al. described ferroptosis as a nonapoptotic, iron-dependent form of cell death that can be triggered by erastin, a lethal small molecule that targets oncogenic ras [66]. Ferroptosis is categorized as an active mode of cell death. It belongs, however, to a class of cell death that resembles sabotage more closely than suicide [67]. In the meantime, different inducers of ferroptosis have been described and their signaling cascades have been elucidated. According to this, ferroptosis proceeds via the inhibition of the cystine/glutamate antiporter [66], leading to depletion of GSH, an increase in oxidative stress and, later on, to the direct or indirect inhibition of glutathione peroxidase 4 (GPX4) [68], which is a lipid repair enzyme that counteracts the generation of lipid hydroperoxides. As a consequence, ferroptosis leads to the accumulation of oxidized lipids and the destruction of cell membrane integrity [69]. Another factor in ferroptosis is Ca^2+^. It has been shown that late in the course of the cell death program, a detrimental Ca^2+^ influx occurs, which is mainly mediated by the Ca^2+^ channel ORAI1 (calcium release-activated calcium channel protein 1 encoded by the *ORAI1* gene) [70]. For example, excess extracellular glutamate either directly or indirectly (via depletion of GSH) leads to an opening of Ca^2+^ channels in the plasma membrane, and the increase in the intracellular Ca^2+^ concentration in turn affects mitochondrial function and metabolism. Indeed, ferroptosis is frequently associated with changes in mitochondrial morphology, bioenergetics, and metabolism [71].

Since erythrocytes are commonly thought of as hemoglobin-filled and, thus, iron-loaded bags encased by a plasma membrane, iron homeostasis should play a major role in the regulation of eryptosis. Indeed, in vitro and in vivo studies revealed that erythrocytes from mice receiving an iron-deficient diet suffer from anemia, which is in part due to higher eryptosis rates and a faster clearance of damaged erythrocytes from the circulation [72]. By contrast, iron overload leads to excessive non-transferrin-plasma iron, which then attacks the plasma membrane, thereby promoting the peroxidative damage of membrane lipids and proteins. In the heart, this results in the clinical manifestation of an often fatal hemosiderotic cardiomyopathy [73]. Recently, it was shown that red blood cell biochemistry is significantly altered in hemochromatosis patients. Here, erythrocytes display significant changes in their membrane composition, as indicated by the phosphatidylserine exposure on the outer leaflet, as well as increased intracellular calpain activity [74]. As far as the signaling components of ferroptosis are concerned, it is interesting that most of the central factors are also found in erythrocytes: (i) erythrocytes contain high levels of GSH and the targeting of GSH leads to eryptosis [20,37]; (ii) the lipid repair enzyme GPX4 is not only expressed in reticulocytes [75] but a similar enzyme activity could also be detected in mature erythrocytes [76]; (iii) Ca^2+^ is one of the main regulators of eryptosis [13,14,15,25], and data from knock-out mice revealed that the impaired regulation of the Ca^2+^ channel ORAI1 leads to compromised cytoskeletal architecture in erythrocytes [77]; and (iv) oxidative stress is a prominent trigger of eryptosis that plays a crucial role in several pathological conditions, such as diabetes and hypercholesterolemia [78,79].

Taken together, there are several overlaps between the ferroptosis of nucleated cells and eryptosis, especially in the specific redox signaling of these unique cell death pathways. However, further research is necessary to unravel the exact role of iron in its different oxidation states in the eryptotic pathway.

## 4. The Role of Eryptosis in Infectious Diseases

Malaria is a disease caused by the protozoan *Plasmodium* spp. that infects erythrocytes. Several groups investigated the role of eryptosis in the progression of this infectious disease [18,26,37,40,80,81,82,83]. According to most studies and the data published so far, infected erythrocytes display increased signs of eryptosis and have a shorter half-life [18,81,82]. Interestingly, several antimalaria drugs induce phosphatidylserine exposure [37,40,80]. These drugs, e.g., dimethyl fumarate or amiodarone, further enhance the removal of infected erythrocytes and prolong the life of *Plasmodium berghei*-infected mice [37,80]. Eryptosis was thus described as an efficient antiparasitic mechanism. However, Boulet et al. recently re-evaluated the experimental conditions of the antimalarial effect of several inducers of erythrocyte programmed cell death. In their work, they concluded that “careful consideration of experimental set up is key for the accurate assessment of the eryptosis-inducing potential of compounds and their evaluation as potential antimalarials” [83]. It should therefore be kept in mind that the antimalarial effect of eryptosis induction has only been demonstrated in animal models and still requires careful clinical evaluation.

Another disease that may involve erythrocytes is pneumonia. Pneumonia can be caused by bacterial, viral or fungal infections of the lungs. A common cause of bacterial pneumonia is *Streptococcus pneumoniae* (pneumococcus). In 2019, a virally induced pneumonia, i.e., severe acute respiratory syndrome coronavirus 2 (SARS-CoV-2) pneumonia, was described. Patients with this disease suffer from respiratory distress, and O_2_ supply to the organs is limited. It was therefore hypothesized that a potential antiviral drug should on the one hand eliminate virus-infected host cells, and, on the other hand, protect the O_2_ transport vehicles of the body, i.e., the red blood cells [84]. In their statement, the authors recommended the use of the specific PKC inhibitor chelerythrine in experimental infection models, as it may drive infected cells into apoptosis and, at the same time, inhibit eryptosis [20,84]. Recently, it was shown in a published case report that the application of the protease inhibitors lopinavir and ritonavir presumably induced eryptosis in a SARS-CoV-2-infected patient [85]. The fact that eryptosis can be detected in clinical settings such as sepsis [86] additionally demonstrates the relevance of this unique cell death pathway during infectious processes.

## 5. The Role of Eryptosis in Hematologic Disorders and Other Diseases

Sickle cell anemia, thalassemia, and glucose-6-phosphate dehydrogenase deficiency are well defined diseases affecting erythrocyte biochemistry. Affected cells have a reduced life span, an effect contributing to anemia. It is thus not surprising that one of the first eryptosis research papers investigated the sensitivity of the erythrocytes of patients suffering from the above-mentioned diseases towards osmotic shock, oxidative stress or energy depletion. In this work, the authors demonstrated that diseased erythrocytes are much more susceptible to stress-induced erythrocyte death, suggesting the pathophysiological role of eryptosis in anemic conditions [16]. In the meantime, these early observations have been repeatedly confirmed for sickle cell anemia [18,58] and thalassemia [87], but also for other hematologic disorders, such as autoimmune hemolytic anemia [56] and antiphospholipid syndrome [88]. In all cases, the pathologic erythrocytes are characterized by enhanced phosphatidylserine exposure.

Erythrocytes are the main cellular constituents of human blood, and the involvement of erythrocytes in the manifestation of atherosclerotic plaque formation has been discussed [89]. Furthermore, several diseases with anemic complications have been associated with enhanced eryptosis. For example, Wilson disease, which is caused by the accumulation of Cu^2+^ in cells, results in liver cirrhosis and anemia. It was shown that Cu^2+^ triggers hepatocyte apoptosis and phosphatidylserine exposure on erythrocytes [34]. Another example is chronic kidney disease, which is frequently accompanied by anemia, hypoxemia, and hypoxia. In this context, the addition of uremic serum from hemodialysis patients aggravates eryptotic signs on otherwise healthy red blood cells [90]. It was therefore concluded that eryptosis represents a biological mechanism responsible for the manifestation of various anemic pathologies [38,47].

## 6. Conclusions

Eryptosis is the premature, stress-induced death of red blood cells, which is distinct from accidental hemolysis or cellular senescence. In particular, the pathways of oxidative stress-induced eryptosis show several similarities to ferroptosis, the iron-dependent death of nucleated cells. Eryptosis can also be found in vivo, and its pathophysiologic relevance has been demonstrated in various diseases and hematologic disorders. Interesting points that might be worth investigating in the future are (i) the direct comparison of eryptosis and programmed cell death in enucleated platelets (ii) the therapeutic potential of eryptosis, which has not yet been exploited. Future studies are therefore needed to demonstrate the relevance of eryptosis signaling for other enucleated cell types, and to show the therapeutic potential of this fundamental death pathway.

## Figures and Tables

**Table 1:** The eryptotic machinery of mature red blood cells.

Protein Name	Function	References
Cation channel	Ca^2+^ entry	Lang et al. [15]
Sphingomyelinase	Formation of ceramide	Lang et al. [32]
Protein kinase C	Protein phosphorylation	Klarl et al. [44]
Nuclear factor κB	Transcription factor ^1^	Ghashghaeinia et al. [60]
Caspase-3	Cysteinprotease	Mandal et al. [50]

^1^ Function in nucleated cells.

## Data Availability

Not applicable.
